# Evaluation of measurement properties of pediatric acute diarrheal severity scoring systems

**DOI:** 10.1186/1745-6215-16-S3-P5

**Published:** 2015-11-24

**Authors:** Samaneh Khanpour Ardestani, Sana Ishaque, Joan Robinson, Levinus A Dieleman, Hien Q Huynh, Hsing Jou, Sunita Vohra

**Affiliations:** 1 Department of Pediatrics, University of Alberta, Edmonton, Canada; 2 School of Population Health, The University of Adelaide, Adelaide, South Australia; 3 Department of Medicine, University of Alberta, Edmonton, Canada

## Background

Interventional studies of pediatric acute diarrhea have used heterogeneous outcome measures, often with poor reporting of their measurement properties. Use of different measures or measures that lack sound measurement properties in trials with similar primary outcomes hampers comparison and knowledge synthesis.

## Objectives

In this systematic review, we evaluated the measurement properties of ten commonly used instruments to assess the severity of acute diarrhea in children.

## Methods

Medline, EMbase and the Cochrane library were searched using a highly sensitive search filter developed by Terwee et al. to identify studies that evaluated measurement properties. This search filter was combined with the names of ten pre-identified scales of pediatric diarrhea severity. Reference lists from included articles and the original publications for the ten diarrhea scales were also reviewed. Eligibility criteria were: 1) ability to develop or evaluate the measurement properties – i.e. content validity, construct validity, reliability or responsiveness – of a measurement instrument; 2) ability to measure severity of diarrhea/gastroenteritis; and 3) ability of the scale to be developed or adapted for the pediatric population (0-18 y/o). The methodological quality of the included studies and the results of measurement properties were appraised using checklists from the COnsensus-based Standards for the selection of health Measurement Instruments (COSMIN) group.

## Results

The search yielded 98 potentially relevant articles, of which only 2 articles met inclusion criteria. Studies that did not evaluate measurement properties of the identified scales or did not measure pediatric diarrhea were excluded. Both included studies evaluated the measurement properties of the “Modified Vesikari score” (MVS). Assessment of methodological quality determined that both studies were of ‘poor’ quality in most properties except for hypothesis testing, which was rated as ‘good’. MVS was rated as positive for face and construct validity and indeterminate for internal consistency and interpretability.

## Conclusion

Despite their wide use, we found a disturbing lack of evidence evaluating validity and reliability of the most commonly used pediatric diarrhea severity scales. Further research with sound methodology is strongly recommended to properly evaluate the measurement properties of these scales. Moreover, to avoid heterogeneity, we encourage researchers to develop scales that measure outcomes identified in a newly developed core outcome set by the COMMENT group for clinical trials in acute diarrhea.

